# Supplementary material for: Angiopoietin-4-dependent venous maturation and fluid drainage in the peripheral retina
Source: eLife. 2018 Nov 16;7:e37776. doi: 10.7554/eLife.37776 (PMC6239434; doi:10.7554/eLife.37776)
Supplement: Supplementary file 1. [file elife-37776-supp1.docx]

**Supplementary file 1. The sequences of primers used for quantitative RT-PCR.**

| moAngpt4ex5For | tccttaaagacacctaagccagtg |
| --- | --- |
| moAngpt4ex6Rev | ggtcctctggaaatttacgcttcc |
| moAngpt4ex8For | atgataactgcatgtgtaaatgtgctc |
| moAngpt4ex9Rev | caggcacccattggcctcagcatc |
| moB-actinFor | tgttaccaactgggacgaca |
| moB-actinRev | ggggtgttgaaggtctcaaa |
| moAngpt1For | cattcttcgctgccattctg |
| moAngpt1Rev | gcacattgcccatgttgaatc |
| moAngpt2For | ttagcacaaaggattcggacaat |
| moAngpt2Rev | ttttgtgggtagtactgtccattca |
| moEphB4For | catcaaggtggacacagtgg |
| moEphB4Rev | aaagagatgcagggagagca |
| moCOUP-TFIIFor | agtactgccgcctcaaaaag |
| moCOUP-TFIIRev | cgttggtcagggcaaact |
| moTie-2For | atgtggaagtcgagaggcgat |
| moTie-2Rev | cgaatagccatccactattgtcc |
| moVegfaFor | acattggctcacttccagaaacac |
| moVegfaRev | ggttggaaccggcatctttatc |
| moMcp-1For | ctgaagccagctctctcttcct |
| moMcp-1Rev | caggcccagaagcatgaca |
| moIcam-1For | cgctgtgctttgagaactgtg |
| moIcam-1Rev | atacacggtgatggtagcgga |
| huTEKFor | ttagccagcttagttctctgtgg |
| huTEKRev | agcatcagatacaagaggtaggg |
| huGAPDHFor | acaactttggtatcgtggaagg |
| huGAPDHRev | gccatcacgccacagtttc |
