# Supplementary material for: Angiopoietin-4-dependent venous maturation and fluid drainage in the peripheral retina
Source: eLife. 2018 Nov 16;7:e37776. doi: 10.7554/eLife.37776 (PMC6239434; doi:10.7554/eLife.37776)
Supplement: Supplementary file 2. [file elife-37776-supp2.docx]

**Supplementary file 2.** **The sequences of primers used for generating *in situ* hybridization probe template plasmids.**

| *in situ* Angpt1For | ataaagcttatgacagttttcctttcctttgcattc |
| --- | --- |
| *in situ* Angpt1Rev | tatggatccgtgaatcttcagaatttcatttgtctgttg |
| *in situ* Angpt2For | ataaagcttatgtggcagatcattttcctaactttt |
| *in situ* Angpt2Rev | tatggatcctttgtagcttgtttatttcactggtct |
| *in situ* Angpt4For | ataaagcttatgctctgccagccagctatgctacta |
| *in situ* Angpt4Rev | tatggatccgccgctgcagctctcggctctgcatca |
| *in situ* Tie2For | tccaagaagcttttctataaacctgtc |
| *in situ* Tie2Rev | taaattgtgggatccggattgttttcg |
